# Fast Phase-Only Positioning with Triple-Frequency GPS

**DOI:** 10.3390/s18113922

**Published:** 2018-11-14

**Authors:** Kan Wang, Pei Chen, Peter J. G. Teunissen

**Affiliations:** 1 Department of Spatial Sciences, Curtin University, Perth 6845, Australia; kan.wang@curtin.edu.au (K.W.); P.Teunissen@curtin.edu.au (P.J.G.T.); 2 School of Astronautics, Beihang University, Beijing 100191, China; 3 Department of Geoscience and Remote Sensing, Delft University of Technology, Delft 2628, The Netherlands

**Keywords:** GPS, phase-only, ambiguity resolution, Ambiguity Dilution of Precision (ADOP), success-rate

## Abstract

In this contribution, we study the phase-only ambiguity resolution and positioning performance of GPS for short baselines. It is well known that instantaneous (single-epoch) ambiguity resolution is possible when both phase and code (pseudorange) data are used. This requires, however, a benign multipath environment due to the severe effects multipath has on the code measurements. With phase-only processing, one would be free from such severe effects, be it that phase-only processing requires a change in receiver-satellite geometry, as a consequence of which it cannot be done instantaneously. It is thus of interest to know how much change in the relative receiver-satellite geometry is needed to achieve successful phase-only ambiguity resolution with correspondingly high precision baseline solutions. In this contribution, we study the two-epoch phase-only performance of single-, dual-, and triple-frequency GPS for varying time spans from 60 s down to 1 s. We demonstrate, empirically as well as formally, that fast phase-only very-precise positioning is indeed possible, and we explain the circumstances that make this possible. The formal analyses are also performed for a large area including Australia, a part of Asia, the Indian Ocean, and the Pacific Ocean. We remark that in this contribution "phase-only" refers to phase-only measurements in the observation model, while the code data are thus only used to compute the approximate values needed for linearizing the observation equations.

## 1. Introduction

In this contribution, we study, empirically and formally, the phase-only ambiguity resolution and positioning performance of single-, dual-, and triple-frequency Global Positioning System (GPS) for short baselines. Phase-only processing, with the purpose of avoiding code multipath, has been studied in [1,2]. In these studies, however, it is not the double-differenced (DD) phase data, but their triple-differenced counterpart that is used. As a consequence, the ambiguities are eliminated from the observation equations, thus making integer ambiguity resolution impossible. In the present contribution, however, we keep the integer ambiguities in the phase-only model and use the change in receiver-satellite geometry to enable ambiguity resolution. This principle was first introduced in [3]. It will be shown in this contribution that, although the DD ambiguities have a very poor precision when the changes in geometry are small, their least-squares ambiguity decorrelation adjustment (LAMBDA)-decorrelated [4] counterparts can still be of sufficient high precision to enable successful ambiguity resolution.

**Figure 1:**
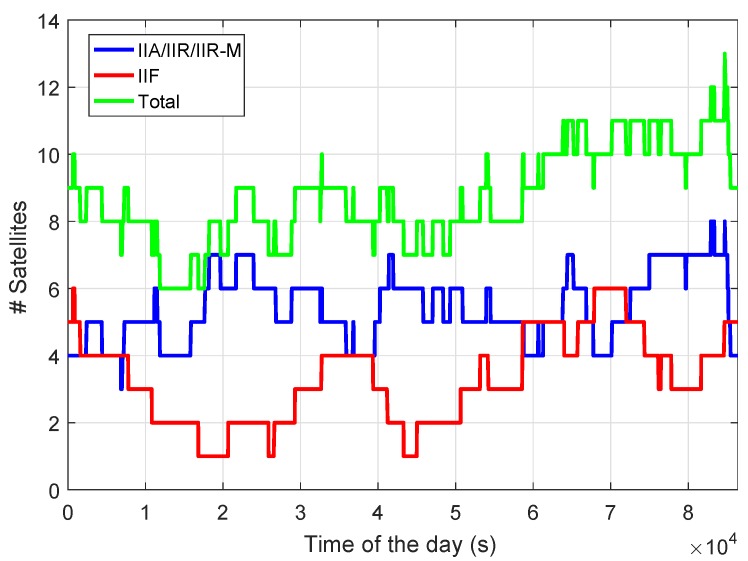
Number of visible GPS satellites for station CUAA in Perth, Australia, on DOY 240, 2018.

By the beginning of September 2018, 31 GPS operational satellites are available consisting of 1 block IIA satellite, 11 block IIR satellites, 7 block IIR-M satellites, and 12 block IIF satellites. The IIF satellites transmit signals on the third frequency L5 in addition to L1 and L2 [5]. Making use of the Multi-GNSS (Global Navigation Satellite System) Experiment (MGEX) broadcast ephemeris [6,7,8] on Day of Year (DOY) 240, 2018, the number of the GPS IIA/IIR/IIR-M satellites transmitting L1 (1575.42 MHz) and L2 (1227.6 MHz) signals, and the number of the GPS IIF satellites transmitting triple-frequency signals on L1, L2, and L5 (1176.45 MHz) are shown in Figure 1 for station CUAA located in Perth, Australia. The elevation mask is set to be 10 degrees in this study. We see that in more than 80% of the time at least 8 GPS satellites can be observed. Around 2–5 IIF satellites are visible most of the time. In this study, all plots are generated using the MGEX broadcast ephemeris on DOY 240, 2018, in GPS time (GPST).

Using the LAMBDA method, in the two-epoch phase-only case, large redundancies are helpful to push down the conditional standard deviations of the decorrelated ambiguities to a low level and thus improve the ambiguity resolution performance [3]. Assuming that the receiver coordinates and the ambiguities remain unchanged, and double-differences are formed on each frequency, the redundancy of the least-squares adjustment can be formulated for ambiguity-float ($R_{f}$) and -fixed cases ($R_{x}$) as

(1) $$\begin{array}{ccc} R_{f} & = & \sum_{j=1}^{f}\left(m_{j}-1\right)-3 \end{array}$$

(2) $$\begin{array}{ccc} R_{x} & = & 2\sum_{j=1}^{f}\left(m_{j}-1\right)-3 \end{array}$$

where $m_{j}$ needs to be larger than 1 to form double-differences on frequency *j*.

In this study, based on single-, dual-, and triple-frequency phase signals of the current GPS constellation, we evaluate the phase-only ambiguity resolution and positioning performance using two epochs separated by different time spans. In the next section, an overview of the processing strategy is given, which is followed by the introduction of our measurement set up and geometry. Subsequently, empirical and formal analysis are performed for the two baselines in Perth, followed by a formal analysis covering a larger area containing Australia, part of the Indian Ocean, the Pacific Ocean, and Asia. Conclusions are provided at the end of the paper.

## 2. Processing Strategy

In a multi-frequency two-epoch scenario, the linearized DD GPS observed-minus-computed (O-C) terms of the phase (Δϕ) observations can be formulated for short baselines as

(3) $$E\left[\begin{array}{c} \Delta \phi (t_{i})\\ \Delta \phi (t_{i}+\Delta t) \end{array}\right]=\left[\begin{array}{cc} D_{m}^{T}A\left(t_{i}\right) & \Lambda \\ D_{m}^{T}A\left(t_{i}+\Delta t\right) & \Lambda \end{array}\right]\left[\begin{array}{c} \Delta b\\ a \end{array}\right]$$

with

(4) $$\begin{array}{ccc} D_{m}^{T} & = & \mathrm{blkdiag}(D_{m_{1}}^{T},\cdots ,D_{m_{f}}^{T}) \end{array}$$

(5) $$\begin{array}{ccc} \Lambda & = & \mathrm{blkdiag}(\lambda_{1}I_{m_{1}-1},\cdots ,\lambda_{f}I_{m_{f}-1}) \end{array}$$

(6) $$\begin{array}{ccc} A(t_{i}) & = & {\left[u^{1}\left(t_{i}\right),\cdots ,u^{m}\left(t_{i}\right)\right]}^{T} \end{array}$$

(7) $$\begin{array}{ccc} m & = & \sum_{j=1}^{f}m_{j} \end{array}$$

where *f* and $m_{j}$ denote the number of frequencies and the number of visible GPS satellites transmitting signals on frequency *j*, respectively. The differencing operator $D_{m_{j}}^{T}=\left[-e_{m_{j}-1},I_{m_{j}-1}\right]$ forms the between-satellite differences with $e_{m_{j}-1}$ and $I_{m_{j}-1}$ denoting the vector of ones and the identity matrix of size $m_{j}-1$, respectively. blkdiag(·) represents the block diagonal matrix of the matrices contained in (·), and E[·] is the expectation operator. The term $u^{j}\left(t_{i}\right)$ represents the unit vector from satellite *j* to the rover at the time point $t_{i}$, and Δt denotes the time span between the two epochs. The wavelength on frequency *j* is denoted by $\lambda_{j}$. The baseline increment vector and the DD ambiguity vector (in cycles) are denoted by Δb and *a*, respectively. Here, we assume the baseline coordinates and the ambiguities remain unchanged during the two epochs. The MGEX broadcast ephemeris [6,7,8] is used to compute the satellite orbits. The two-epoch processing is only performed when, during the two epochs, the visible satellites are the same and no cycle slips occur during the two epochs. We remark that in this contribution "phase-only" refers to phase-only measurements in the observation model (Equation (3)). Before the two-epoch processing, the GPS L1 code observations were used in a single point positioning (SPP) procedure to obtain the satellite positions at the signal transmitting time.

Based on the zenith-referenced phase signal standard deviations on each frequency *j*, denoted as $\sigma_{j}$, the variance-covariance matrix of Equation (3) reads

(8) $$D\left[\begin{array}{c} \Delta \phi (t_{i})\\ \Delta \phi (t_{i}+\Delta t) \end{array}\right]=\left[\begin{array}{cc} 2D_{m}^{T}Q\left(t_{i}\right)D_{m} & 0\\ 0 & 2D_{m}^{T}Q\left(t_{i}+\Delta t\right)D_{m} \end{array}\right]$$

with

(9) $$\begin{array}{ccc} Q(t_{i}) & = & \mathrm{blkdiag}(Q_{1}\left(t_{i}\right),\cdots ,Q_{f}\left(t_{i}\right)) \end{array}$$

(10) $$\begin{array}{ccc} Q_{j}\left(t_{i}\right) & = & \sigma_{j}^{2}W_{j}^{-1}\left(t_{i}\right) \end{array}$$

(11) $$\begin{array}{ccc} W_{j}\left(t_{i}\right) & = & \mathrm{diag}(w^{j,1}\left(t_{i}\right),\cdots ,w^{j,m_{j}}\left(t_{i}\right)) \end{array}$$

where D[·] is the dispersion operator, and diag(·) forms the diagonal matrix with the diagonal elements contained in (·). The $w^{j,s}\left(t_{i}\right)$ represents the elevation-dependent weight of the *s*-th satellite transmitting signals on frequency *j*, which is formulated as [9]

(12) $$w^{j,s}\left(t_{i}\right)={\left(1+10\cdot \exp\left(-\frac{e^{j,s}\left(t_{i}\right)}{10}\right)\right)}^{-2}$$

where exp(·) is the natural exponential function. The term $e^{j,s}\left(t_{i}\right)$ denotes the elevation angle from receiver to the *s*-th satellite transmitting signals on frequency *j* at $t_{i}$, which is given in degrees.

Using the least-squares variance component estimation (LS-VCE) procedure [10], the zenith-referenced standard deviations were calculated for two baselines CUAA-CUBB and CUAA-CUCC in Perth, Australia, on frequencies L1, L2, and L5 for GPS phase measurements (Table 1). The phase standard deviations were calculated based on the DD phase residuals computed using the ground truth of the baselines and the reference ambiguities, which were obtained with the strong baseline-known model [11,12]. The data on DOY 241, 2018, were used for the computation. Note that the phase multipath was not corrected for the signal standard deviations. In this study, the phase signals on channels L1C, L2W, and L5X (see Table A.5 of [13], p.1211) were used for the signal analysis and data processing.

## 3. Measurement Geometry

**Figure 2:**
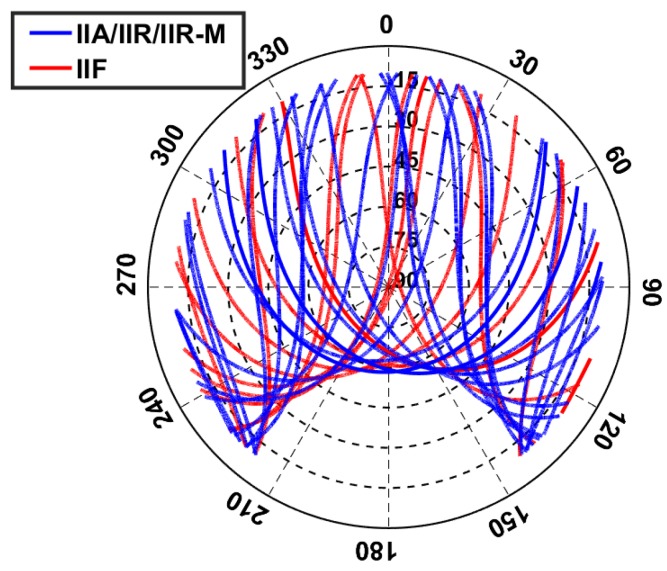
Skyplot of the visible GPS satellites for station CUAA over 24 h on DOY 240, 2018.

In this study, 1 Hz GPS data of two meter-level short baselines CUAA-CUBB and CUAA-CUCC located in Perth, Australia, were collected for the data processing. The three stations CUAA, CUBB, and CUCC are all equipped with Javad receivers (Javad, San Jose, CA, U.S.) of the type JAVAD TRE_G3TH DELTA and Trimble antennas (Trimble, Sunnyvale, CA, U.S.) of the same type TRM59800.00 SCIS. The skyplot for station CUAA is shown as an example in Figure 2 for all the visible GPS satellites above the elevation mask of 10${}^{\circ }$.

**Figure 3:**
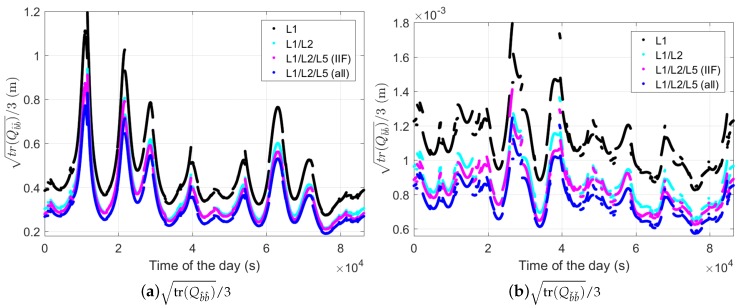
$\sqrt{\mathrm{tr}(Q_{\hat{b}\hat{b}})}/3$ (**a**) and $\sqrt{\mathrm{tr}(Q_{\hat{b}\hat{b}})}/3$ (**b**) in the two-epoch case with Δt of 30 s. The satellite orbit on DOY 240, 2018, and the ground truth of baseline CUAA-CUBB were used for the plot.

Based on [14], in the two-epoch phase-only case, the variance-covariance matrix of the ambiguity-float ($Q_{\hat{b}\hat{b}}$) and -fixed baseline increments ($Q_{\overset{ˇ}{b}\overset{ˇ}{b}}$) at $t_{i}$ can be formulated as

(13) $$\begin{array}{ccc} Q_{\hat{b}\hat{b}} & = & 2{\left(\sum_{k=1}^{2}{\left(D_{m}^{T}A_{k}-\bar{A}\right)}^{T}P_{k}\left(D_{m}^{T}A_{k}-\bar{A}\right)\right)}^{-1} \end{array}$$

(14) $$\begin{array}{ccc} Q_{\overset{ˇ}{b}\overset{ˇ}{b}} & = & 2{\left(\sum_{k=1}^{2}A_{k}^{T}D_{m}P_{k}D_{m}^{T}A_{k}\right)}^{-1} \end{array}$$

with

(15) $$\begin{array}{ccc} \bar{A} & = & {\left(\sum_{k=1}^{2}P_{k}\right)}^{-1}\sum_{k=1}^{2}\left(P_{k}D_{m}^{T}A_{k}\right) \end{array}$$

(16) $$\begin{array}{ccc} P_{k} & = & {\left(D_{m}^{T}Q\left(t_{i}+\left(k-1\right)\Delta t\right)D_{m}\right)}^{-1} \end{array}$$

where the subscript k=1,2 represent the time point $t_{i}$ and $t_{i}+\Delta t$. As the average precision in all three directions, Figure 3 shows the terms $\sqrt{\mathrm{tr}(Q_{\hat{b}\hat{b}})}/3$ and $\sqrt{\mathrm{tr}(Q_{\overset{ˇ}{b}\overset{ˇ}{b}})}/3$ in single-, dual-, and triple-frequency cases for baseline CUAA-CUBB with Δt of 30 s, where tr(·) is the trace operator. From Figure 3 it can be observed that increasing the number of frequency from one (black lines) to three (magenta lines) reduces the values of $\sqrt{\mathrm{tr}(Q_{\hat{b}\hat{b}})}/3$ and $\sqrt{\mathrm{tr}(Q_{\overset{ˇ}{b}\overset{ˇ}{b}})}/3$ by around 20–30%. Very similar values between the dual- and triple-frequency cases (the cyan and magenta lines) are caused by the fact that only block IIF satellites transmit signals on L5. In the case that L5 is transmitted by all available GPS satellites (see the blue lines), increasing the frequency number from two to three will reduce the average precision by around 10% from their values in the dual-frequency case. We remark that the uncontinuities observed in Figure 3b also exist in Figure 3a. They can be observed by zooming in on the figure.

**Figure 4:**
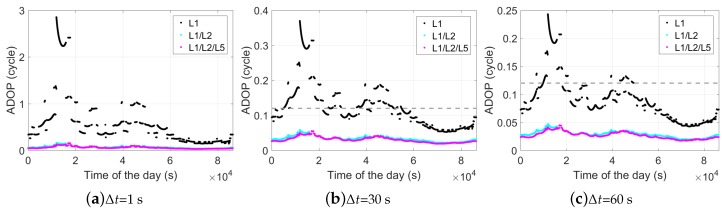
Ambiguity dilution of precision (ADOP) of baseline CUAA-CUBB in the phase-only two-epoch case. The time span between the two epochs is 1 (**a**), 30 (**b**), and 60 s (**c**). The gray dahsed lines mark the ADOPs of 0.12 cycles. Note that the sub-figures have different scales

As introduced in [15], the ambiguity dilution of precision (ADOP) is an easy-to-calculate scalar that measures the model strength of successful ambiguity resolution. It can be computed as

(17) $$\mathrm{ADOP}={\sqrt{|Q_{\hat{a}\hat{a}}|}}^{\frac{1}{m-f}}$$

where $Q_{\hat{a}\hat{a}}$ denotes the variance-covariance matrix of the float ambiguities, and |·| is the determinant operator. When increasing the number of frequencies and the time span, the ADOPs are correspondingly changed as shown in Figure 4. We see that the ADOPs are highly dependent on the time span Δt, i.e., the geometry change between the two epochs. However, even with a Δt of 1 s, in the dual- and triple-frequency cases, the ADOPs are smaller than 0.12 cycles in above 90% of all tested two-epoch cases. For Δt of 30 and 60 s, the ADOPs in the dual- and triple-frequency cases are below 0.12 cycles (the gray dashed lines) in all tested two-epoch cases. As described in [16], an ADOP lower than 0.12 cycles approximately corresponds to an integer least-squares (ILS) ambiguity success rate (ASR) larger than 99.9%, which is lower bounded by the integer bootstrapping (IB) ASR [17]. This indicates high ASRs in the dual- and triple-frequency cases without needing to wait for a long time to collect the second epoch of the phase data.

The ADOP can be related to the baseline precision before ($Q_{\hat{b}\hat{b}}$) and after ($Q_{\overset{ˇ}{b}\overset{ˇ}{b}}$) ambiguity resolution by means of the gain numbers [14]. The gain numbers $\gamma_{k}$, (*k* = 1, 2, 3) are defined as

(18) $$\gamma_{k}\left(f_{k}\right)=\frac{f_{k}^{T}Q_{\hat{b}\hat{b}}f_{k}}{f_{k}^{T}Q_{\overset{ˇ}{b}\overset{ˇ}{b}}f_{k}}$$

where the 3×1 vectors $f_{k}$ are called gain vectors. The stationary values of the gain numbers $\gamma_{k}$ with $\gamma_{1}\leq \gamma_{2}\leq \gamma_{3}$ are generalized eigenvalues of $Q_{\hat{b}\hat{b}}$ and $Q_{\overset{ˇ}{b}\overset{ˇ}{b}}$, which fulfill

(19) $$|Q_{\hat{b}\hat{b}}-\gamma_{k}Q_{\overset{ˇ}{b}\overset{ˇ}{b}}|=0$$

Based on [16], the determinant of the variance-covariance matrix of the ambiguities $Q_{\hat{a}\hat{a}}$ can be formulated as

(20) $$|Q_{\hat{a}\hat{a}}|=\frac{|{\left(\sum_{k=1}^{2}P_{k}/2\right)}^{-1}|}{|\Lambda^{2}|}\times \frac{|Q_{\hat{b}\hat{b}}|}{|Q_{\overset{ˇ}{b}\overset{ˇ}{b}}|}=\frac{|{\left(\sum_{k=1}^{2}P_{k}/2\right)}^{-1}|}{|\Lambda^{2}|}\times \prod_{k=1}^{3}\gamma_{k}$$

**Figure 5:**
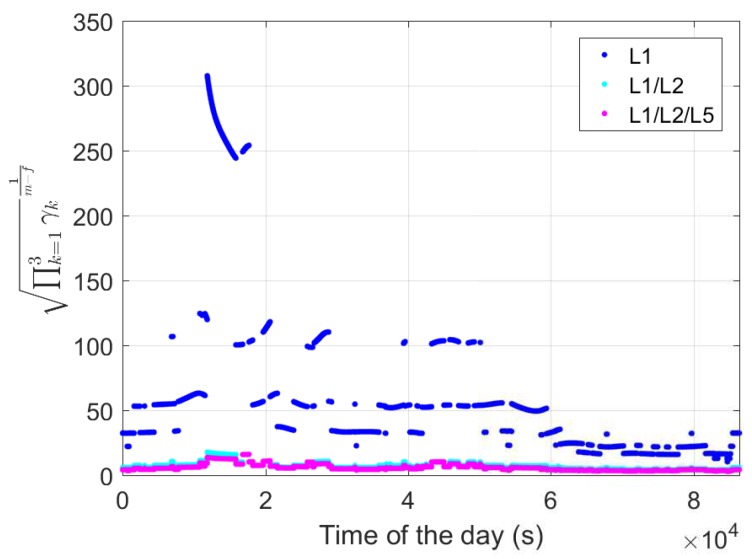
The term ${\sqrt{\prod_{k=1}^{3}\gamma_{k}}}^{\frac{1}{m-f}}$ (Equation (20)) for baseline CUAA-CUBB with Δt of 1 s.

As ADOP is equal to ${\sqrt{|Q_{\hat{a}\hat{a}}|}}^{\frac{1}{m-f}}$ (Equation (17)), the outliers that we see in ${\sqrt{\prod_{k=1}^{3}\gamma_{k}}}^{\frac{1}{m-f}}$ for the L1-only case between around 11900 and 17700 s, as shown in Figure 5, explain the ADOP outliers in the L1-only case during the same time periods (Figure 4a).

**Figure 6:**
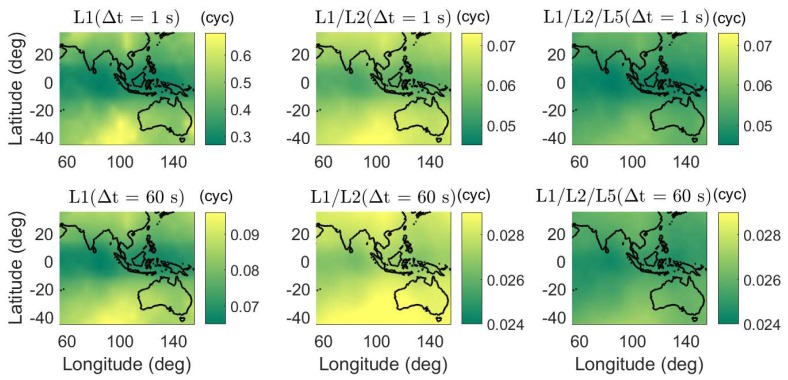
Daily average ADOPs using single-, dual-, and triple-frequency phase signals with Δt of 1 (top) and 60 s (bottom). Note that the sub-figures have different scales.

In Figure 6, the daily average ADOPs are illustrated for short baselines from 55${}^{\circ }$ E to 155${}^{\circ }$ E in longitude and from 45${}^{\circ }$ S to 35${}^{\circ }$ N in latitude. The average ADOPs in the colormaps were computed based on two-epoch time series with a sampling interval of 30 s. The signal standard deviations of the baseline CUAA-CUBB (Table 1) were used for the computation. We see that, in dual- and triple-frequency cases, even with Δt of 1 s, the average ADOPs are smaller than 0.12 cycles in the entire test area.

## 4. Ambiguity Resolution

In this section, the performance of ambiguity resolution is evaluated for the phase-only two-epoch scenario. Making use of the two baselines introduced in Section 3 and the phase standard deviations given in Table 1, different frequency combinations and time spans between the two epochs are tested for both formal and empirical analysis.

In this study, we use the LAMBDA method [4] to decorrelate the float ambiguities, which were estimated in Equation (3) together with the baseline increments. As given in [18], using the conditional standard deviations of the *decorrelated* ambiguities $\sigma_{{\hat{z}}_{i|I}}$ with i=1,⋯,m−f and I=1,⋯,i−1, the IB ASR, denoted as $P_{IB}$, can be calculated as

(21) $$P_{IB}=\prod_{i=1}^{m-f}\left(2\Phi \left(\frac{1}{2\sigma_{{\hat{z}}_{i|I}}}\right)-1\right)$$

with

(22) $$\Phi \left(x\right)=\int_{-\infty }^{x}\frac{1}{\sqrt{2\pi }}\exp\left(-\frac{y^{2}}{2}\right)dy.$$

**Figure 7:**
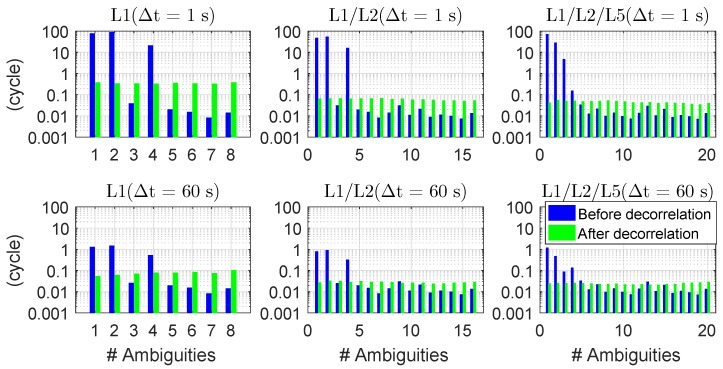
Conditional standard deviations of the ambiguities ($\sigma_{{\hat{a}}_{i|I}}$ before the decorrelation and $\sigma_{{\hat{z}}_{i|I}}$ after the decorrelation with I=1,⋯,i−1) in single-, dual-, and triple-frequency cases for phase-only two-epoch processing. The satellite orbit on DOY 240, 2018, and the ground truth of baseline CUAA-CUBB were used for the plot. The first epoch is the first second of the test day, and the time span between the two epochs are 1 (**top**) and 60 s (**bottom**).

As shown in [3], without decorrelation of the ambiguities, some of the conditional standard deviations of the original ambiguities $\sigma_{{\hat{a}}_{i|I}}$ are large in the phase-only two-epoch case, especially when the time span between the two epochs is small and almost no geometry change exists between the two epochs. This leads to difficult search of the integer ambiguities. After decorrelating the ambiguities, the situation is changed with the conditional standard deviations reduced to a relative low and equal level. As shown in Figure 7, based on two epochs of data for baseline CUAA-CUBB on DOY 240, 2018, the conditional standard deviations of the ambiguities before (blue) and after (green) decorrelation are shown for single-, dual-, and triple-frequency cases. The first epoch is the first second of the test day, and the time span between the two epochs is set to be 1 and 60 s in the top and bottom panels, respectively. It can be observed that for a short time span of 1 s between the two epochs (see the top panels of Figure 7), the largest conditional standard deviations amounting to tens of cycles without decorrelation are reduced to the level of centi- to deci-cycles. With a longer time span of 60 s between the two epochs, with a larger geometry change, the conditional standard deviations are reduced both before and after decorrelation of the ambiguities. The conditional standard deviations in dual- and triple-frequency scenarios are in general smaller than those in single-frequency scenario.

**Figure 8:**
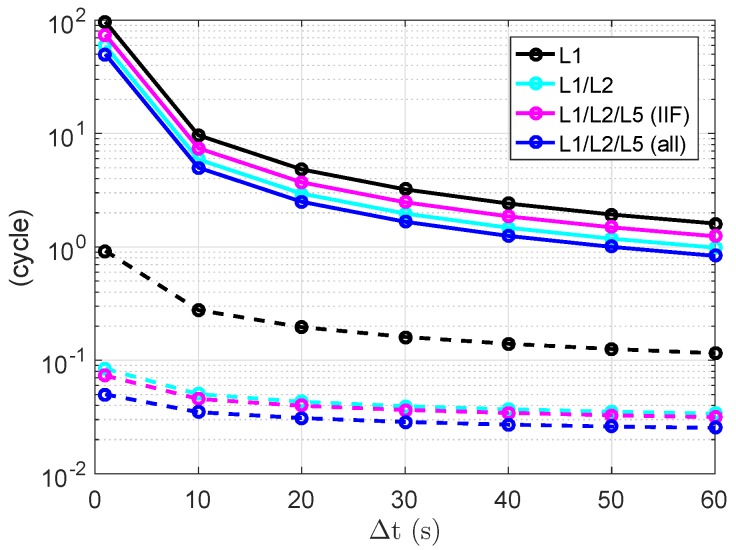
The terms ${\bar{\sigma }}_{\bar{a}}$ (Equation (25)) in solid lines and ${\bar{\sigma }}_{\bar{z}}$ (Equation (26)) in dashed lines. The satellite orbit on DOY 240, 2018, and the ground truth of baseline CUAA-CUBB were used for the plot.

To have an overview of the conditional standard deviations on different time spans between the two epochs, we compute the average of the largest three conditional standard deviations of the original and decorrelated ambiguities for each two-epoch case, denoted as $\sigma_{\bar{a}}\left(t_{i}\right)$ and $\sigma_{\bar{z}}\left(t_{i}\right)$, respectively, which are formulated as

(23) $$\begin{array}{ccc} \sigma_{\bar{a}}\left(t_{i}\right) & = & \sqrt{\frac{\sigma_{{\hat{a}}_{1}^{\max}}^{2}\left(t_{i}\right)+\sigma_{{\hat{a}}_{2}^{\max}}^{2}\left(t_{i}\right)+\sigma_{{\hat{a}}_{3}^{\max}}^{2}\left(t_{i}\right)}{3}} \end{array}$$

(24) $$\begin{array}{ccc} \sigma_{\bar{z}}\left(t_{i}\right) & = & \sqrt{\frac{\sigma_{{\hat{z}}_{1}^{\max}}^{2}\left(t_{i}\right)+\sigma_{{\hat{z}}_{2}^{\max}}^{2}\left(t_{i}\right)+\sigma_{{\hat{z}}_{3}^{\max}}^{2}\left(t_{i}\right)}{3}} \end{array}$$

where $\sigma_{{\hat{a}}_{k}^{\max}}\left(t_{i}\right)$ and $\sigma_{{\hat{z}}_{k}^{\max}}\left(t_{i}\right)$ represent the *k*-th largest conditional standard deviation of the original and decorrelated ambiguities at $t_{i}$, respectively. The daily average of these values using all tested two-epoch cases on the test day is defined as

(25) $$\begin{array}{ccc} {\bar{\sigma }}_{\bar{a}} & = & \sqrt{\frac{\sum_{i}^{N}\sigma_{\bar{a}}^{2}\left(t_{i}\right)}{N}} \end{array}$$

(26) $$\begin{array}{ccc} {\bar{\sigma }}_{\bar{z}} & = & \sqrt{\frac{\sum_{i}^{N}\sigma_{\bar{z}}^{2}\left(t_{i}\right)}{N}} \end{array}$$

where *N* stands for the number of the tested two-epochs cases within the day, which is larger than 78,600 for all tested frequency combinations and time spans in this study. Figure 8 shows these ${\bar{\sigma }}_{\bar{a}}$ and ${\bar{\sigma }}_{\bar{z}}$ in solid and dashed lines, respectively. We see that the large conditional standard deviations are significantly reduced after decorrelating the ambiguities. After decorrelating the ambiguities (dashed lines), the reduction is more significant when changing from single- to dual-frequency processing, and when increasing the Δt from 1 to 10 s. Making an assumption that all GPS satellites send L5 signals (see the blue lines), the ${\bar{\sigma }}_{\bar{a}}$ and ${\bar{\sigma }}_{\bar{z}}$ can be further reduced in the triple-frequency case.

In Table 2, both the empirical and average formal IB ASRs are given for different frequency combinations and time spans. The empirical ASR $P_{E}$ is calculated as

(27) $$P_{E}=\frac{N_{C}}{N}$$

where $N_{C}$ represents the number of tested two-epoch cases with ambiguities correctly fixed. We see that for a short time span of 1 and 10 s in the single-frequency case, the empirical and the average formal ASR do not correspond well with each other. This is caused by the fact that the formal ASRs are very sensitive to the phase signal standard deviations in such cases. Note that the phase signal standard deviations given in Table 1 are rounded values and amount to around 1.0, 1.3, and 1.5 mm on L1, L2, and L5 for CUAA-CUBB. Increasing them by sub-millimetres could, e.g., lead to reduction in the average formal ASRs to around 0.2 for Δt of 1 s in the L1-only case. At the same time, it can also be observed that the ASRs in dual- and triple-frequency cases are almost 100% even for a short time span of 1 s. Using GPS dual-frequency signals on L1 and L2, or triple-frequency signals on L1, L2, and L5, ambiguities can be quickly resolved when collecting 2 s of phase data.

**Figure 9:**
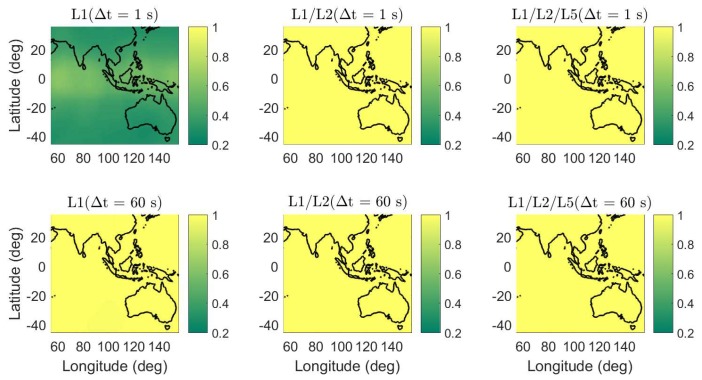
Average formal IB ASRs on single-, dual-, and triple-frequencies in the phase-only two-epoch case. The time span between the two epochs are 1 (**top**) and 60 s (**bottom**).

Apart from the baselines in Perth, the average formal IB ASRs are also computed for short baselines from 55${}^{\circ }$ E to 155${}^{\circ }$ E in longitude and from 45${}^{\circ }$ S to 35${}^{\circ }$ N in latitude (Figure 9). The signal standard deviations of baseline CUAA-CUBB (Table 1) were used for the processing. The average ASRs in the colormaps were computed based on two-epoch time series with a sampling interval of 30 s. The time span Δt between the two epochs are set to be 1 and 60 s, respectively. As for the baselines in Perth, the formal average ASRs in dual- and triple-frequency cases are high even with a short Δt of 1 s, i.e., above 0.99 in the entire test area.

## 5. Positioning Performance

Using the 1 Hz phase data of the two baselines introduced in Section 3, the baseline increments are estimated in both the ambiguity-float and -fixed cases. The precision of the ambiguity-float and -fixed baseline increments will be discussed in Section 5.1 and Section 5.2.

### 5.1. Ambiguity-Float Solutions

**Figure 10:**
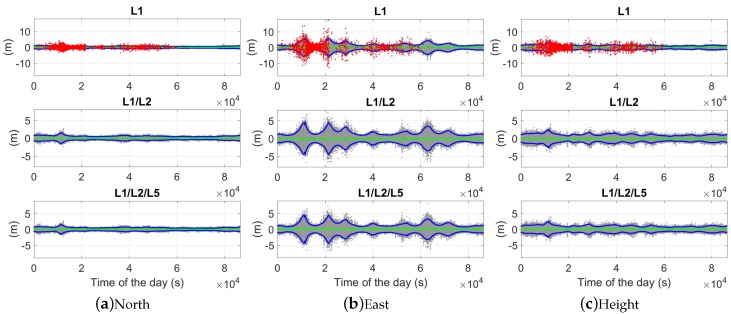
Baseline errors for CUAA-CUBB in the phase-only two-epoch case. The time span between the two epochs is 30 s. The gray, green, and red dots represent the ambiguity-float, -correctly-fixed, and -wrongly-fixed solutions, and the blue dots represent the 95% formal confidence intervals of the ambiguity-float solutions. Note that the scales in different sub-figures are different.

In Figure 10, the baseline errors are shown in the north-, east-, and up-directions in the phase-only two-epoch case with a time span of 30 s. The gray and blue dots illustrate the ambiguity-float baseline errors and their 95% formal confidence intervals, and the green and red dots represent the solutions with ambiguities correctly and wrongly fixed, respectively. The frequently appearing red dots from around 0.5 ×$10^{4}$ to 2.2 ×$10^{4}$ s and from around 3.6 ×$10^{4}$ to 5.2 ×$10^{4}$ s in the L1-only case also correspond to the relatively high ADOP (above 0.12 cycles) during these time periods in Figure 4b. In dual- and triple-frequency cases, with the time span between the two epochs of 30 s, the ambiguity-float baseline errors are within 1 m in all three directions in about 75% of the time.

**Figure 11:**
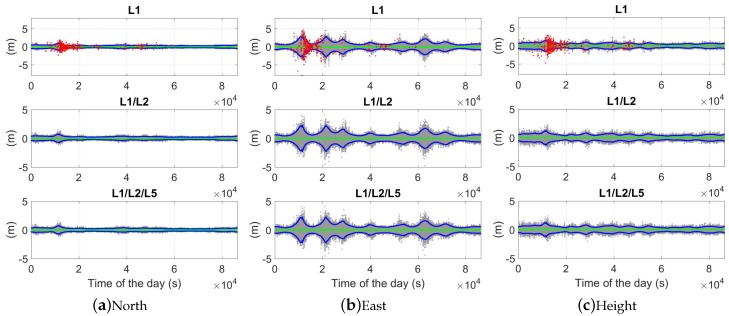
Baseline errors for CUAA-CUBB in the phase-only two-epoch case. The time span between the two epochs is 60 s. The gray, green, and red dots represent the ambiguity-float, -correctly-fixed, and -wrongly-fixed solutions, and the blue dots represent the 95% formal confidence intervals of the ambiguity-float solutions. Note that the scales in different sub-figures are different.

Using the time span of 60 s instead, as shown in Figure 11, fewer red dots appear in the top panels of the L1-only case. This corresponds to the fact that the L1-only ADOPs larger than 0.12 cycles in Figure 4c are less than those in Figure 4b. The red dots from around 1 ×$10^{4}$ to 2 ×$10^{4}$ s and from around 4.3 ×$10^{4}$ to 4.9 ×$10^{4}$ s also correspond to the time periods with ADOPs larger than 0.12 cycles (Figure 4c).

**Figure 12:**
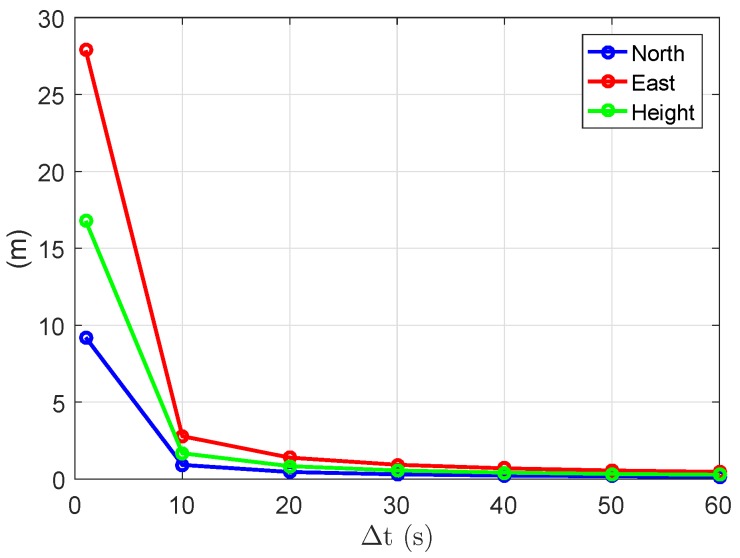
Average formal standard deviations of the baseline errors in the ambiguity-float case. GPS triple-frequency phase data of baseline CUAA-CUBB was used for the plots.

Table 3 lists the empirical and average formal (in brackets) standard deviations of the ambiguity-float baseline errors. The average formal standard deviation is defined as the square root of the average formal variances in all tested two-epoch cases. We see that increasing the number of frequencies does not lead to dramatic changes in the ambiguity-float positioning performance, but the length of the time span is essential for the positioning precision. Taking the triple-frequency case as an example, as shown in Figure 12, the average formal standard deviations show dramatic changes when varying the time span from 1 to 10 s. For both baselines in the tested single-, dual-, and triple-frequency scenarios, with a time span of 10 s, the average formal standard deviations are within meter level in all three directions. The values are further reduced to within or around 1 m when increasing the time span to 30 s. From Table 3, we also see that the average formal and empirical standard deviations of the baseline errors (Table 3) have shown certain differences when the time span between the two epochs is short. This can be explained by the fact that the weak model with almost no geometry changes between the two epochs is more sensitive to the model deficiency by calculating the signal standard deviations. As the real signal noise containing phase multipath does not perfectly follow the elevation-dependent weighting function (Equation (12)), slight differences could result between the formal signal standard deviations given in Table 1 and the real data. The influences of these differences on the ambiguity-float baseline errors are related to the model strength.

**Figure 13:**
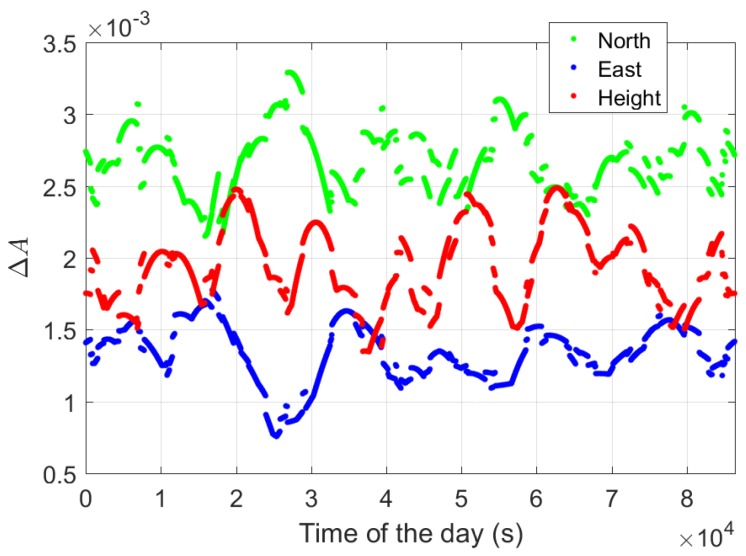
Average change of $A_{k}$ (Equation (28)) in the north-, east-, and up-directions. The time span between the two epochs is 1 s. Data of baseline CUAA-CUBB was used for the plot.

As shown by Equation (13), the ambiguity-float baseline variance-covariance matrix $Q_{\hat{b}\hat{b}}$ is highly related to the time change of $A_{k}$, which is reflected in $A_{k}-\bar{A}$. This explains the fact that the standard deviations of the baseline errors are sensitive to the time span Δt between the two epochs in the ambiguity-float case (Table 3). Additionally, in the ambiguity-float case, we observe that the east precision is worse than those in the other two directions (Figure 10 and Figure 11, Table 3). This is related to the fact that the GPS satellites moves in general slower in the west–east direction than in the other two directions for our baselines. Figure 13 shows the average change of $A_{k}$ in all three directions in the L1-only case, which is defined for each two-epoch case as

(28) $$\Delta A=\frac{\sum_{s=1}^{m}\left|A_{2}^{s}-A_{1}^{s}\right|}{m}$$

where $A_{1}^{s}$ and $A_{2}^{s}$ denote the *s*-th row of the matrices $A_{1}$ and $A_{2}$, respectively. From Figure 13, we see that the smallest time change can be achieved in the east direction, while the largest time change happens in the north direction. This explains the poorest baseline precision in the east direction, and the best precision in the north direction (Table 3).

**Figure 14:**
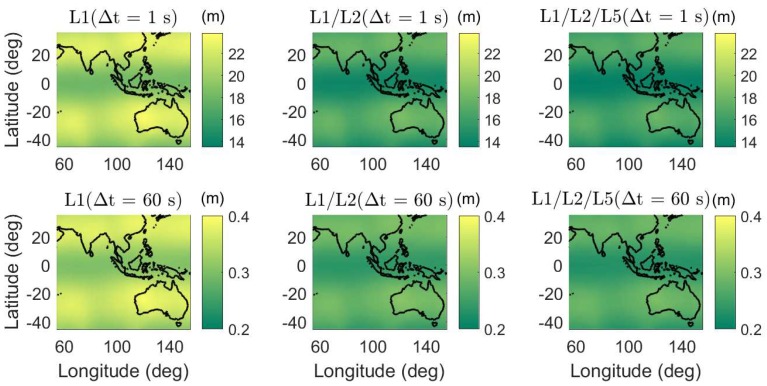
Average formal standard deviations of ambiguity-float heights in the phase-only two-epoch case. The time span between the two epochs are 1 (**top**) and 60 s (**bottom**). Note that the scales of the sub-figures are different.

The average formal standard deviations of the ambiguity-float baseline errors are also computed for short baselines in a large area from 55${}^{\circ }$ E to 155${}^{\circ }$ E in longitude and from 45${}^{\circ }$ S to 35${}^{\circ }$ N in latitude. The signal standard deviations of baseline CUAA-CUBB (Table 1) were used for the processing. The height components of the average formal standard deviations are given as examples in the ambiguity-float case in Figure 14. As for the baselines in Perth (Table 3), for a short time span of 1 s, the standard deviations of the baseline errors are at the level of tens of meters, while they are reduced to dm-level when the time span is increased to 60 s.

### 5.2. Ambiguity-Fixed Solutions

**Figure 15:**
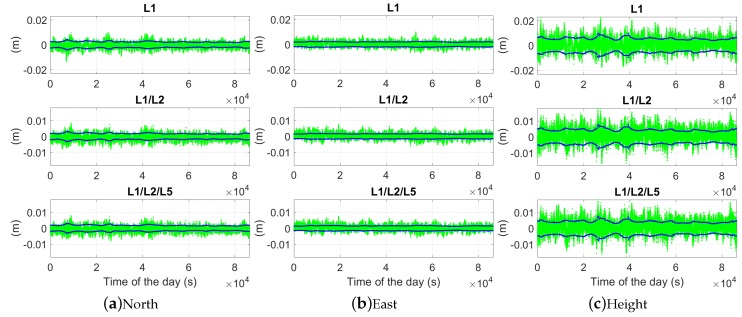
Ambiguity-fixed baseline errors for CUAA-CUBB in the phase-only two-epoch case. The time span between the two epochs is 30 s. The green dots represent the ambiguity-correctly-fixed solutions, and the blue dots represent their 95% formal confidence intervals. Note that the scales in different sub-figures are different.

With Figure 10 zoomed in, the ambiguity-fixed solutions and their 95% formal confidence intervals are illustrated for the same baseline CUAA-CUBB with the same time span of 30 s between the two epochs (Figure 15). In dual- and triple-frequency cases, the correctly fixed ambiguity solutions are within 1 cm in the north and east directions in 100% of the time, and in the height directions in above 98% of the time.

**Figure 16:**
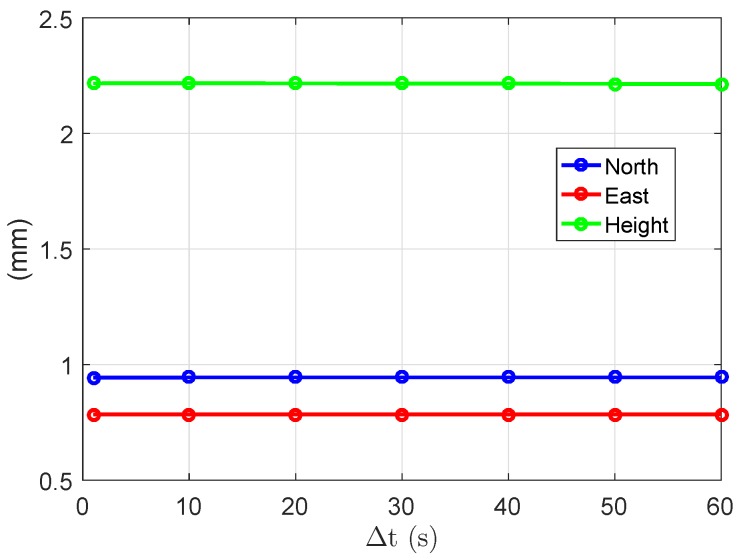
Average formal standard deviations of the baseline errors in the ambiguity-fixed case. GPS triple-frequency phase data of baseline CUAA-CUBB was used for the plots.

The empirical and average formal standard deviations of the baseline errors are also given in Table 4 in the ambiguity-fixed case. Only the two-epoch cases with ambiguities successfully fixed contribute to the ambiguity-fixed standard deviations. The L1-only standard deviations of the ambiguity-fixed solutions are not shown for the time span of 1 s due to the low ASR (Table 2). As shown in Table 4 and Figure 16, compared to the ambiguity-float case, the standard deviations of the ambiguity-fixed baseline errors do not vary much with the time spans between the two epochs. The models with different time spans also become almost equally sensitive to the differences between the formal phase signal standard deviations and the real data. In dual- and triple-frequency cases, the standard deviations of the ambiguity-fixed baseline errors are at the mm-level even with a short Δt of 1 s.

**Figure 17:**
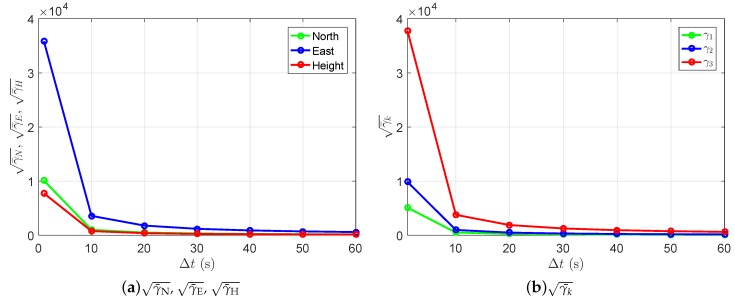
Square roots of the daily average $\gamma_{N}$, $\gamma_{E}$, $\gamma_{H}$ (**a**, Equation (29)), and $\gamma_{k}$ (**b**, Equation (19)). The L1 data of baseline CUAA-CUBB on DOY 240, 2018, was used for the plot.

After fixing the ambiguities, the formal precision improvement of the baseline errors in the north- ($\gamma_{N}$), east- ($\gamma_{E}$), and up-directions ($\gamma_{H}$) can be defined as

(29) $$\gamma_{N}=\frac{\sigma_{\hat{N}}^{2}}{\sigma_{\overset{ˇ}{N}}^{2}},\;\gamma_{E}=\frac{\sigma_{\hat{E}}^{2}}{\sigma_{\overset{ˇ}{E}}^{2}},\;\gamma_{H}=\frac{\sigma_{\hat{H}}^{2}}{\sigma_{\overset{ˇ}{H}}^{2}}$$

where $\sigma_{\hat{N}}$, $\sigma_{\hat{E}}$ and $\sigma_{\hat{H}}$ represent the formal standard deviation of the north, east, and up baseline increments in the ambiguity-float case, and $\sigma_{\overset{ˇ}{N}}$, $\sigma_{\overset{ˇ}{E}}$, and $\sigma_{\overset{ˇ}{H}}$ represent those in the ambiguity-fixed case. Based on Equation (18), $\gamma_{N}$, $\gamma_{E}$, and $\gamma_{H}$ are gain numbers in the north-, east-, and up-directions with the gain vectors $f_{N}={\left[1,0,0\right]}^{T}$, $f_{E}={\left[0,1,0\right]}^{T}$, and $f_{H}={\left[0,0,1\right]}^{T}$. Figure 17a shows the square roots of the average precision gain over the entire day ($\sqrt{{\bar{\gamma }}_{N}}$, $\sqrt{{\bar{\gamma }}_{E}}$, and $\sqrt{{\bar{\gamma }}_{H}}$) in the L1-only case. We see that the largest improvement can be achieved in the east direction, and in all three directions, the improvements are more significant at short time spans. Note that, compared to the amplitudes of the improvements, the differences between the single-, dual-, and triple-frequency scenarios are not significant.

The stationary values of the gain numbers, which fulfill Equation (19), also vary with the time spans between the two epochs. Figure 17b shows the square roots of the daily average gain numbers ($\sqrt{{\bar{\gamma }}_{k}}$) in the L1-only case. We see that, on a daily average, the largest precision gain in 3-dimensional space is around 3.8 ×$10^{4}$ at the short time span of 1 s. The value of $\sqrt{{\bar{\gamma }}_{3}}$ (red line) is reduced to around 600 at the time span of 60 s. The dual- and triple-frequency scenarios show similar patterns as in the single-frequency case.

**Figure 18:**
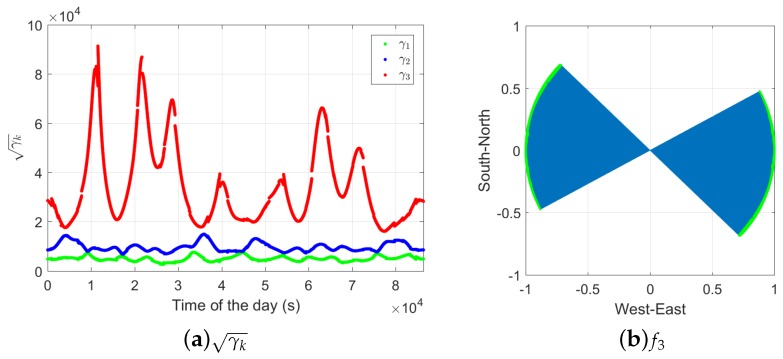
Square roots of the stationary values of the gain numbers (**a**, Equation (19)) and the corresponding gain vectors $f_{3}$ in the horizontal plane (**b**). Data of baseline CUAA-CUBB is used for the L1-only case. The time span between the two epochs is 1 s.

In Figure 18a, the square roots of these gain numbers ($\sqrt{\gamma_{k}}$) are shown for DOY 240, 2018, and baseline CUAA-CUBB in the L1-only case with the time span Δt of 1 s. We see that the largest daily value of $\sqrt{\gamma_{3}}$ has reached more than 9 ×$10^{4}$. The gain vector $f_{3}$ indicates the direction in which the largest precision gain can be achieved. Figure 18b shows the $f_{3}$ for the same baseline on DOY 240, 2018, in the horizontal plane. The vectors start at the point (0,0) and end at the positions of the green dots. The height components are not shown since they are small, i.e. within ±0.2 during the entire day. In Figure 18b, we see that the largest precision improvement is mainly achieved in the west–east direction. This corresponds to the fact shown in Figure 17a that among the three directions (north, east, and up), the east precision has achieved the largest improvement after ambiguity fixing.

**Figure 19:**
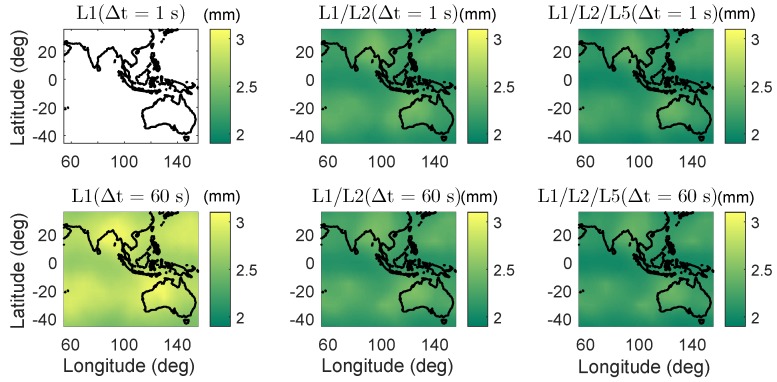
Average formal standard deviations of ambiguity-fixed heights in the phase-only two-epoch case. The time span between the two epochs are 1 (**top**) and 60 s (**bottom**).

The average formal standard deviations of the height errors in the ambiguity-fixed case are shown in Figure 19. Note that only the two-epoch cases with a formal ASR larger than 0.999 contribute to the average formal standard deviations here. In the L1-only case with Δt of 1 s (left top panel of Figure 19), the ambiguity-fixed solutions are not plotted because of the low ASRs. As for the baselines in Perth, in the ambiguity-fixed cases, the standard deviations of the baseline errors are at the mm-level in the dual- and triple-frequency cases.

## 6. Conclusions

To avoid the code multipath effects in GNSS processing, in this study, phase-only GPS phase measurements of two epochs were used to resolve the integer ambiguities and to estimate the baseline errors in ambiguity-float and -fixed cases. Using different frequency combinations and time spans between the two epochs, the ambiguity resolution and positioning performances were evaluated with 1 Hz data collected from two baselines in Perth, Australia, based on both formal and empirical analysis. Formal analysis is also performed for a large area covering Australia, part of the Indian Ocean, the Pacific Ocean, and Asia. We remark that in this contribution "phase-only" refers to phase-only measurements in the observation model, while the code data are thus only used to compute the approximate values needed for linearizing the observation equations.

Based on the empirical and formal analysis of the two baselines in Perth and the signal standard deviations used for these two baselines, using dual- and triple-frequency GPS signals, high ASRs can be achieved even when the time span between the two epochs is as short as 1 s. In the ambiguity-float case, the standard deviations of the baseline errors are highly dependent on the time span between the two epochs. In L1-only, L1/L2-combined, and the triple-frequency cases, with a time span of 10 s, the standard deviations of the ambiguity-float baseline errors are at meter-level. The standard deviations are further reduced to within or around 1 m, when the time span is increased to 30 s. In the ambiguity-fixed case, the standard deviations of the baseline errors are at the mm-level in dual- and triple-frequency cases.

Formal analysis was also performed for a large area including Australia, part of the Indian Ocean, the Pacific Ocean, and Asia. High ASRs can be obtained in dual- and triple-frequency cases for a short time span of 1 s, and the ambiguity-fixed positioning precision is at the mm-level in dual- and triple-frequency cases.

## Figures and Tables

**Table 1:** Zenith-referenced phase standard deviations for baselines CUAA-CUBB and CUAA-CUCC.

Frequency	CUAA-CUBB (mm)	CUAA-CUCC (mm)
L1	1	1
L2	1	2
L5	2	2

**Table 2:** Empirical and average formal IB ASRs (in brackets) for the phase-only two-epoch scenario. The data on DOY 240, 2018, was used for the computation.

Frequency	CUAA-CUBB			CUAA-CUCC		
	1 s	10 s	60 s	1 s	10 s	60 s
L1	0.186(0.317)	0.695(0.823)	0.966(0.989)	0.185(0.303)	0.677(0.808)	0.961(0.987)
L1/L2	0.988(0.999)	1.000(1.000)	1.000(1.000)	0.974(0.998)	1.000(1.000)	1.000(1.000)
L1/L2/L5	0.996(1.000)	1.000(1.000)	1.000(1.000)	0.988(0.999)	1.000(1.000)	1.000(1.000)

**Table 3:** Empirical and average formal (in brackets) standard deviations of the ambiguity-float baseline errors for the phase-only two-epoch scenario. The data on DOY 240, 2018, was used for the computation. The results are given in the format of the north/east/up directions.

Frequency	CUAA-CUBB (m)		CUAA-CUCC (m)	
	1 s	60 s	1 s	60 s
L1	7(12)/23(37)/14(22)	0.2(0.2)/0.6(0.6)/0.4(0.4)	7(13)/23(38)/14(23)	0.2(0.2)/0.6(0.6)/0.4(0.4)
L1/L2	8(10)/24(29)/15(17)	0.2(0.2)/0.5(0.5)/0.3(0.3)	7(11)/24(32)/15(19)	0.2(0.2)/0.5(0.5)/0.3(0.3)
L1/L2/L5	8(9)/25(28)/15(17)	0.2(0.2)/0.5(0.5)/0.3(0.3)	7(10)/24(31)/15(18)	0.2(0.2)/0.5(0.5)/0.3(0.3)

**Table 4:** Empirical and average formal (in brackets) standard deviations of the ambiguity-fixed baseline errors for the phase-only two-epoch scenario. The data on DOY 240, 2018, was used for the computation. The results are given in the format of the north/east/up directions.

Frequency	CUAA-CUBB (mm)		CUAA-CUCC (mm)	
	1 s	60 s	1 s	60 s
L1	–	2(1)/2(1)/4(3)	–	2(1)/2(1)/4(3)
L1/L2	2(1)/2(1)/4(2)	2(1)/1(1)/4(2)	2(1)/2(1)/4(3)	2(1)/2(1)/4(3)
L1/L2/L5	2(1)/2(1)/4(2)	2(1)/1(1)/4(2)	2(1)/2(1)/4(2)	2(1)/1(1)/3(2)
